# Reduction of endotoxicity in Bordetella bronchiseptica by lipid A engineering: Characterization of lpxL1 and pagP mutants

**DOI:** 10.1080/21505594.2021.1929037

**Published:** 2021-05-31

**Authors:** Jesús Pérez-Ortega, Roel M. Van Harten, Ria Van Boxtel, Michel Plisnier, Marc Louckx, Dominique Ingels, Henk P. Haagsman, Jan Tommassen

**Affiliations:** a Section Molecular Microbiology, Department of Biology, Faculty of Science, Utrecht University, Utrecht, Netherlands; b Institute of Biomembranes, Utrecht University, Utrecht, Netherlands; c Section of Molecular Host Defense, Division of Infectious Diseases and Immunology, Department of Biomolecular Health Sciences, Faculty of Veterinary Medicine, Utrecht University, Utrecht, Netherlands; d GSK, Rixensart, Belgium

**Keywords:** *Bordetella*, LPS, LpxL1, PagP, TLR4, endotoxicity, vaccine, antimicrobial susceptibility, autoaggregation, biofilms

## Abstract

Whole-cell vaccines against Gram-negative bacteria commonly display high reactogenicity caused by the endotoxic activity of lipopolysaccharide (LPS), one of the major components of the bacterial outer membrane. Underacylation of the lipid A moiety of LPS has been related with reduced endotoxicity in several Gram-negative species. Here, we evaluated whether the inactivation of two genes encoding lipid A acylases of *Bordetella bronchiseptica*, i.e. *pagP* and *lpxL1*, could be used for the development of less reactogenic vaccines against this pathogen for livestock and companion animals. Inactivation of *pagP* resulted in the loss of the secondary palmitate chain at position 3' of lipid A, but hardly affected the potency of the LPS to activate the Toll-like receptor 4 (TLR4). Inactivation of *lpxL1* resulted in the loss of the secondary 2-hydroxy laurate group present at position 2 of lipid A and, unexpectedly, in the additional loss of the glucosamines that decorate the phosphate groups at positions 1 and 4' and in an increase in LPS molecules carrying O-antigen. The resulting LPS showed greatly reduced potency to activate TLR4 in HEK-Blue reporter cells expressing human or mouse TLR4 as well as in porcine macrophages. Characterization of the *lpxL1* mutant revealed many pleiotropic phenotypes, including increased resistance to SDS and rifampicin, increased susceptibility to cationic antimicrobial peptides, decreased auto-aggregation and biofilm formation, and a tendency to decreased infectivity of macrophages, which are all related to the altered LPS structure. We suggest that the *lpxL1* mutant will be useful for the generation of safer vaccines.

## Introduction

Lipopolysaccharide (LPS), also known as endotoxin, is a major component of the outer membrane of Gram-negative bacteria. It contains a lipid A moiety, which anchors the molecule into the lipid bilayer, and a core oligosaccharide attached to the lipid A. In many species, LPS also contains a polysaccharide, known as O-antigen, with a strain-specific sugar composition. Lipid A is responsible for the endotoxic activity of LPS. It is recognized by the Toll-like receptor 4 (TLR4) and the co-receptor myeloid differentiation-2 (MD-2) of innate immune cells, which then triggers the production of pro-inflammatory cytokines, such as TNF-α and IL-1β. This response is essential for clearing local infections, but an overreaction can cause serious damage, including septic shock and death [1].

Lipid A generally consists of a glucosamine disaccharide backbone substituted with phosphates at 1 and 4ʹ positions and acylated at the 2, 2ʹ, 3 and 3ʹ positions with fatty acids. These acyl chains bear β-hydroxyl groups that allow for secondary acylation. The biosynthesis pathway of lipid A, known as the Raetz pathway, is conserved, yet differences in lipid A structure among species do exist. Furthermore, several lipid A variants may coexist in the same species and/or emerge under different environmental conditions [2]. Structural variation, resulting from the regulation of gene expression, may be introduced during the biosynthesis or through post-synthetic modification systems, and includes (de-)acylation, (de-)phosphorylation, or substitution of the phosphates with groups, such as 4-amino-4-deoxy-L-arabinose (L-Ara4N), glucosamine, or phosphoethanolamine [2]. Endotoxicity is mainly determined by the presence or absence and the substitution of the phosphate groups along with the number, length and distribution of the acyl chains [2,3]. For instance, decrease in the number of fatty acids or phosphate groups is commonly related to diminished endotoxic responses.

*Bordetella bronchiseptica* is a Gram-negative bacterium that causes respiratory diseases in several mammalian species, such as infectious tracheobronchitis in dogs (a.k.a. kennel cough) or atrophic rhinitis in pigs [4]. In addition, it has been related to zoonotic infections in immunocompromised and healthy humans in close contact with infected animals [5]. Prophylaxis is ordinarily achieved through vaccination, although booster doses seem to be necessary to keep long-term protection [6]. Adverse reactions to whole-cell vaccines cause animal-welfare problems in livestock and companion animals and necessitate the development of new veterinary vaccines. Although undesired effects of whole-cell vaccines against *B. bronchiseptica* have been poorly studied, they have been a concern for the last decades [6,7]. *B. bronchiseptica* LPS is a very potent TLR4 agonist that produces stronger inflammatory responses than that of *B. pertussis* in vitro and in vivo [8]. To reduce the potential for adverse reactions, parenteral acellular formulations and intranasal vaccines were developed. Nevertheless, several studies showed that parenteral whole-cell vaccines produce faster, higher and more enduring antibody responses than the acellular and intranasal preparations [6]. By genetic engineering of the vaccine strain, changes in the lipid A structure can be induced to moderate TLR4 activation and minimize the risk of whole-cell vaccine reactogenicity.

**Figure 1.**
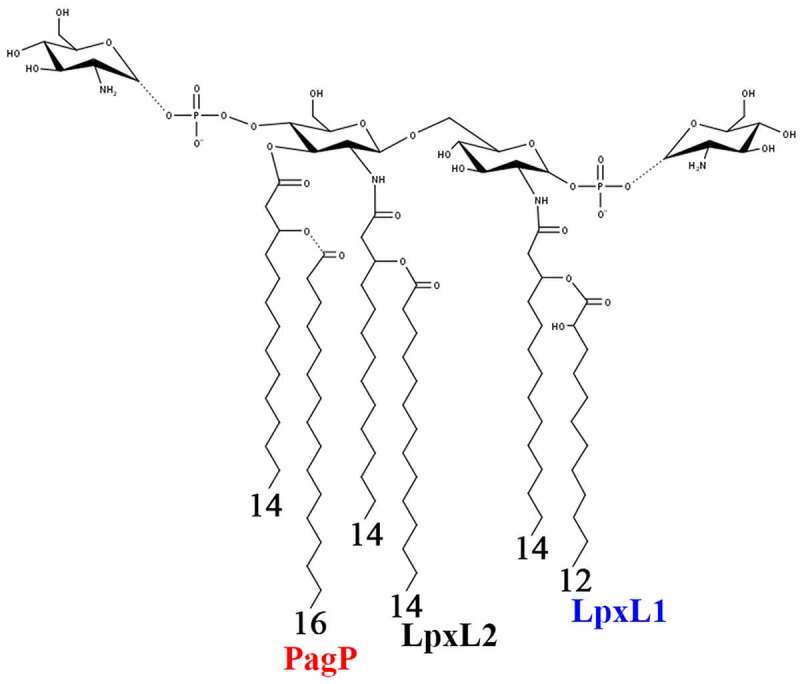
Representation of the lipid A structure of *B. bronchiseptica*. The non-stoichiometric glucosamine and C_16_ acyl chain modifications are denoted by dashed bond lines. The enzymes responsible for the addition of the secondary acyl chains are indicated

*B. bronchiseptica* lipid A is generally known as a hexa-acylated structure with three primary acyl chains, each substituted with a secondary acyl chain (Figure 1). The primary acyl chain at the 3 position is lacking due to the post-synthetic activity of the deacylase PagL [9]. However, several studies have shown extensive intraspecies variability in the ratio of hexa- to penta-acylated molecules [9–11]. Two of the secondary acyl chains are introduced during the biosynthetic pathway by the acyl transferases LpxL1 and LpxL2. Homologues of these enzymes have been identified in *Bordetella pertussis* [12], but their role in *B. bronchiseptica* has not been studied yet. In *B. pertussis*, LpxL2 mediates the introduction of a secondary myristate (C_14_) at the 2ʹ position of the lipid A structure, whereas LpxL1 is responsible for the addition of a secondary 2-hydroxy-laurate (2-OH C_12_) in the 2 position (Figure 1). However, the latter was only observed when the *lpxL1* gene was overexpressed, adding an extra acyl chain to the normally penta-acylated lipid A structure. This modification resulted in higher TLR4 activation compared to the wild type [12]. In contrast, this 2-OH C_12_ chain is present in the lipid A of wild-type *B. bronchiseptica*, suggesting full activity of the LpxL1 enzyme in this species [9,10]. The hydroxylation commonly present in this fatty acid is dependent on the hydroxylase activity of an enzyme encoded by another gene, *lpxO* [9].

The third secondary acyl chain is a palmitate (C_16_), which is attached at the 3ʹ position by the outer membrane-based enzyme PagP (Figure 1). Expression of the *pagP* gene is regulated by the major virulence-regulatory system BvgAS and acylation takes place in the Bvg^+^ phase [13]. PagP-mediated acylation is reported to protect *B. bronchiseptica* against antibody-mediated complement lysis, which increases its persistence during respiratory infection [13,14]. At present, modulation of endotoxic activity by PagP has not been studied in *B. bronchiseptica*. However, the recombinant expression of the *pagP* gene in *B. pertussis*, where expression is otherwise abolished due to the insertion of an *IS* element in the promoter region, led to palmitoylation of the lipid A molecule and to increased TLR4 activation in macrophages stimulated with the LPS [15].

Another modification featured among *Bordetella* spp. is the substitution of the phosphate groups with glucosamine (Figure 1). LgmB-mediated addition of glucosamine is induced in the Bvg^+^ phase in *B. bronchiseptica* and *B. pertussis* [16–18]. In *B. pertussis*, this modification appears to be highly relevant for TLR4 activation in human but not in murine macrophages [19]. Accordingly, *lgmB* inactivation in *B. bronchiseptica* did not affect TLR4 stimulation in murine macrophages [17], but it decreased the resistance of the bacteria to cationic antibacterial peptides.

With the eventual goal to construct a vaccine strain with limited reactogenicity, we investigated in this study the effect of *pagP* and *lpxL1* inactivation in *B. bronchiseptica* on lipid A structure and their consequences on TLR4 activation. We also explored the impact of the mutations on various phenotypes of the bacteria.

## Materials and methods

### Bacterial strains and growth conditions

All bacterial strains used in this study are described in Supplementary Table S1. *Escherichia coli* strains were grown at 37°C in lysogeny broth (LB) while shaking or on LB agar plates. *B. bronchiseptica* strains were grown at 35°C on Bordet-Gengou agar (Difco) supplemented with 15% defibrinated sheep blood (Biotrading) (BG-blood). For liquid cultures, the bacteria were inoculated from plate in Verwey medium [20] and grown at 35°C while shaking at 175 rpm. Alternatively, a Stainer-Scholte (SS) medium [21] was used which was supplemented with 14 g/l Bacto casamino acids (BD biosciences) and then adjusted to pH 7.6. After overnight growth, the optical density at 600 nm (OD_600_) was adjusted to 0.1, and cultures were grown for the time indicated. When needed for plasmid maintenance or strain selection, the following antibiotics were included in the medium: cefotaxime (5 µg/ml), streptomycin (300 µg/ml), gentamicin (10 µg/ml), ampicillin (100 µg/ml for *E. coli*, 200 µg/ml for *B. bronchiseptica*).

### DNA manipulation and construction of mutants

All plasmids and PCR primers used in this study are listed in Supplementary Tables S1 and S2, respectively. Regular PCR reactions were performed using DreamTaq DNA polymerase (Thermo Scientific), whilst PCR fragments generated for cloning were obtained using the Expand High Fidelity PCR system (Roche Diagnostics GmbH). For purification of PCR products, the commercial Wizard SV Gel and PCR Clean-Up System (Promega) was employed. Plasmids were isolated with the E.Z.N.A. Plasmid Mini Kit I (Omega Bio-Tek). PCR products and plasmids were digested with the appropriate restriction enzymes (Thermo Scientific) according to manufacturer’s instructions, purified, and ligated using T4 DNA ligase (5 U/µl) (Thermo Scientific).

To inactivate the *pagP* gene in *B. bronchiseptica*, we replaced the locus on the chromosome by a gentamicin-resistance cassette (*gem^R^*). DNA fragments upstream (920 bp) and downstream (905 bp) of *pagP* were amplified by PCR from strain BB-P19, using a single colony from plate as DNA template. PCR products were introduced separately in the pCRII vector following the instructions of the manufacturer (Invitrogen), yielding plasmids pCRII-PagPup and pCRII-PagPdw, respectively. Upstream and downstream fragments were then combined into a single vector after digestion of both plasmids with endonucleases Eco81I and XbaI, obtaining pCRII-PagPup-PagPdw. A *gem^R^* cassette was amplified from pYRC and inserted in between the two fragments after digestion of construct and PCR product with Eco81I, generating pCRII-PagPup-GemR-PagPdw. Hitherto, the DNA cloning was performed in *E. coli* strain DH5α by transformation using the CaCl_2_ method. The construct obtained was then sub-cloned into the suicide vector pKAS32 after digestion of both plasmids with EcoRI, yielding pKAS-PagPup-GemR-PagPdw. The plasmid obtained was used to transform *E. coli* strain SM10(λpir) to allow for its replication and subsequent transfer to *B. bronchiseptica* by conjugation for inactivation of the chromosomal gene by allelic exchange. First, transconjugants were selected on plates containing gentamicin as well as cefotaxime for counter selection against *E. coli*. Subsequently, selection was made with streptomycin and gentamicin to ensure the loss of plasmid backbone together with the wild-type *pagP* gene.

To inactivate the *lpxL1* gene, the *lpxL1* locus was amplified by PCR including 748 bp upstream of the ATG start codon and 315 bp downstream of the stop codon, and the PCR product was introduced into the pCRII vector resulting in plasmid pCRII-upLpxL1dw. Then, the *gem^R^* cassette was inserted into a PfoI site within the *lpxL1* coding sequence to disrupt the locus (pCRII-upLpxL1dw-GemR). The construct was subcloned into the pKAS32 vector after digestion with SacI and XbaI, yielding pKAS-upLpxL1dw-GemR, which was then used to inactivate the chromosomal gene in *B. bronchiseptica* as above.

### LPS purification and analysis

LPS was isolated from bacterial cells grown in Verwey medium using the Tri-Reagent method [22]. Briefly, lyophilized cells were resuspended in TRIzol Reagent (Invitrogen) by intensive vortexing. After 10 min incubation at room temperature to allow for complete cell homogenization, 20 µl of chloroform (HPLC grade) per mg of cells were added. After vigorous vortexing, the mixture was incubated at room temperature for another 10 min and, subsequently, centrifuged for 10 min to separate phases. The aqueous phase was collected and three additional extractions were performed by adding Milli-Q water to the organic phase, vortexing, incubation at room temperature and centrifugation. All the aqueous phases collected were combined, and the water was evaporated using a speed vacuum concentrator. The pellet was washed with 0.375 M MgCl_2_ in 95% ethanol, pelleted again by centrifugation, and resuspended in Milli-Q water. The extracted LPS was lyophilized, weighed for quantification, and resuspended in endotoxin-free HyPure cell culture grade water (HyClone) for further use. The purity and integrity of purified samples were evaluated by sodium dodecyl sulfate-polyacrylamide gel electrophoresis (SDS-PAGE) combined with silver staining of LPS [23] or Coomassie staining of proteins.

Lipid A was isolated as previously described [24], with slight modifications. Briefly, lyophilized LPS was dissolved in 100 mM ammonium acetate pH 4.5, heated at 80°C for 2 h in 1% SDS and lyophilized overnight. Then, lipid A was suspended in water and washed three times with acidified ethanol to remove the detergent. Subsequently, it was solubilized in chloroform:methanol (2:1, v/v), dried under nitrogen stream, and finally solubilized again in methanol:water (1:1, v/v). Lipid A was analyzed by liquid chromatography–mass spectrometry (LC-MS) in the negative-ion mode [25].

### TLR4 stimulation assays

HEK-Blue TLR4 cells co-expressing either human or murine TLR4, MD-2, and CD14 genes and an NF-κB-inducible secreted embryonic alkaline phosphatase (SEAP) reporter (Invivogen) were cultured according to the manufacturer’s instructions. For TLR4 activation, 2.5 × 10^4^ cells/well were incubated in a 96-well plate with serial dilutions of either purified LPS or whole bacterial cells previously killed by heat treatment at 56°C for 1 h. After 17 h of incubation at 37°C in a 5% saturated CO_2_ atmosphere, supernatants were collected and incubated with 1 mg/ml of the SEAP substrate *p*-nitrophenyl phosphate in 1 M diethanolamine substrate buffer (pH 9.8) for 1 h, and the absorbance at 405 nm was measured in a Biotek microplate reader. To analyze the LPS content of the heat-killed cells used in the assay, the cells were mixed 1:1 with 2x concentrated SDS-PAGE sample buffer, boiled for 5 min, and incubated for 1 h at 65°C with 50 µg/ml of proteinase K (Thermo Scientific). The samples were then analyzed on SDS-PAGE gels with 14% acrylamide and the gels were stained with silver [23].

### Porcine bone marrow-derived macrophages (PBMMs)

PBMMs were grown as previously described [26]. Briefly, bone marrow was harvested from eight young adult pigs by puncture of the pelvis, from which mononuclear cells were isolated by Ficoll (GE Healthcare) density gradient centrifugation and subsequently frozen for storage. To differentiate toward macrophages, cells were cultured in RPMI medium (Gibco) supplemented with 10% fetal calf serum (Corning) for 6 days exposed to either 1% granulocyte macrophage colony-stimulating factor (GM-CSF) (Bio-Rad) for pro-inflammatory M1 macrophages or 30 ng/ml macrophage colony-stimulating factor (M-CSF) (Peprotech) for anti-inflammatory M2 macrophages. Expression of M1 and M2 surface markers was determined by flow cytometry [26]. All animals were used and kept under the approval and guidelines of the animal ethical committee of Utrecht University.

To quantify cytokine production, M1 macrophages were stimulated with 10 ng/ml of purified LPS for 24 h. Then, the supernatant was collected and stored at −20°C until further use. For cytokine quantification, porcine-specific ELISAs (R&D Systems) were performed according to the manufacturer’s recommendations.

To analyze the bacterial survival upon incubation with macrophages, bacteria were grown for 4 h in Verwey medium. Then, the bacterial cells were washed and resuspended in RPMI supplemented with 10% fetal calf serum. Mature porcine macrophages were exposed to the bacteria for 4 h at a multiplicity of infection (MOI) of 1 and, after removal of the supernatant, lysed with 1% Triton X-100 in phosphate-buffered saline (PBS). To measure internalized bacteria, the cells were exposed to 150 µg/ml colistin sulfate (Sigma Aldrich) in PBS for 1 h at 37°C and then washed before lysis. Samples from supernatant, lysate (attached + internalized bacteria), or antibiotic-treated lysate (internalized bacteria) were plated on BG-blood agar and incubated for 72 h at 37°C, and colonies were counted. Total growth was calculated from the colony-forming units (CFU) in the supernatant plus those in the lysate. Attached bacteria were calculated from the CFU in the lysate minus those in the lysate of cells treated with antibiotics. Data obtained with macrophages from one specific pig was identified as outlier using the ROUT test (GraphPad) with 1% aggressiveness and discarded.

### Sensitivity to SDS and antimicrobials

Bacterial cultures were grown in Verwey medium for 4 h, and the OD_600_ was adjusted to 0.1 in fresh medium. Samples of 180 µl of this culture were incubated with 20 µl of either 10% SDS or, as a control, Milli-Q water at 35°C in static conditions. After 2 h, 10-fold serial dilutions were prepared, and drops of 10 µl were spotted on BG-blood agar plates and incubated for 2 days at 35°C.

Minimal inhibitory concentrations (MICs) of different antibiotics were determined by Etest (BioMerieux). Bacterial cultures were grown to an OD_600_ of 0.6 in Verwey medium, and 200-µl samples of the cultures were spread on BG-blood agar in square Petri dishes and allowed to dry for 30 min. Etest strips were placed on the plates, which were then incubated for 2 days at 35°C before establishing MICs from the zone of growth inhibition.

### Settling experiments and biofilm formation

For settling experiments, cultures grown for 48 h in SS medium supplemented with casamino acids were adjusted to an OD_600_ of 1 and incubated in test tubes at room temperature in static conditions. Samples from the top of the tubes were taken for OD_600_ measurements.

For biofilm formation, cultures grown for 24 h in SS medium supplemented with casamino acids were adjusted to an OD_600_ of 0.5. Then, 1-ml samples of the cultures were incubated for 24 h under static conditions at 35°C in 24-well plates. To quantify the biofilm, supernatants were removed, the wells were washed with physiological salt solution, and biofilms were stained for 2 min with 1 ml of 0.5% crystal violet. After two washes with physiological salt solution, the stained biofilm was resuspended in 500 µl of 33% (v/v) acetic acid in water and then quantified by OD_630_ measurements of appropriate dilutions. The values measured for dilutions were multiplied with the dilution factor to obtain the OD_630_ of the original solution.

### Bacterial adhesion to hydrocarbons (BATH)

BATH assay was performed as described [27]. Cultures were grown for 20 h in Verwey medium and bacterial cells were washed and resuspended in PBS, and the concentration was adjusted to an OD_600_ of 1. To test tubes containing 1.5 ml of bacterial solution, 1 ml of hexadecane was either added or not, and the suspensions were vigorously vortexed for 2 min. Aqueous and organic phases were allowed to separate for 15 min at room temperature, and the OD_600_ was measured from samples taken from the bottom of the tube (aqueous phase). BATH (%) was expressed as (n − h)/n × 100, where n is the OD_600_ in the tube without hexadecane and h is the OD_600_ from the tube with hexadecane.

### Outer membrane isolation and analysis

To isolate outer-membrane fractions, overnight grown 25-ml cultures of *B. bronchiseptica* were inactivated by incubation for 1 h at 56°C and the OD_600_ was determined. Cells were harvested by centrifugation at 10,000 rpm (Eppendorf 5920-R centrifuge, FA-6 × 50 rotor) for 5 min and washed with physiological salt solution. The cells were resuspended in 1 ml of 0.75 M sucrose, 10 mM Tris–HCl (pH 7.8). Then, 3 μl of 40 mg/ml lysozyme were added and 2 ml of 1.5 mM EDTA (pH 7.8). The suspension was incubated for 30 min at room temperature. Then, the cell preparations were diluted to OD_600_ of 5 by the addition of a solution of 0.25 M sucrose, 3.33 mM Tris–HCl, 1.0 mM EDTA (pH 7.8). The cells were frozen at − 80°C, thawed, and lysed by ultrasonication. Unbroken cells and aggregates were removed by centrifugation for 1 h at 10,000 rpm in an Eppendorf 5920-R centrifuge (FA-6 × 50 rotor). The supernatant was then centrifuged for 1 h at 50,000 rpm (Beckman Coulter Optima TLX-120 K, TLA 100.2 rotor), and the resulting pellet containing the outer membranes was resuspended in water.

Outer membrane proteins were separated by SDS-PAGE and stained with Bradford reagent as described [28]. Alternatively, the separated proteins were transferred to a 0.45-μm pore-size nitrocellulose membrane (GE Healthcare). For immunodetection, mouse antiserum directed against autotransporter BrkA, generously provided by Nathalie Devos (GlaxoSmithKline Biologicals SA) and rabbit antisera directed against the major porin OmpP and siderophore receptor FauA [29] were used. As secondary antibodies, horseradish peroxidase-conjugated goat anti-mouse or anti-rabbit IgG antisera (ThermoFisher) were employed. Membranes were developed with the Clarity Western ECL Blotting Substrate (Bio-Rad).

### Statistical analysis

All statistical analyses were performed using the GraphPad Prism software (versions 6 and 8). For curve comparison, data from settling and TLR4 stimulation assays were used to calculate the mean area under the curve and standard error of the mean (SEM). In all cases, data was analyzed for statistical significance using one-way ANOVA (Dunnett’s correction for multiple comparison). In addition, a repeated measures design was used during studies with macrophages.

## Results

### Lipid A structural analysis

**Figure 2.**
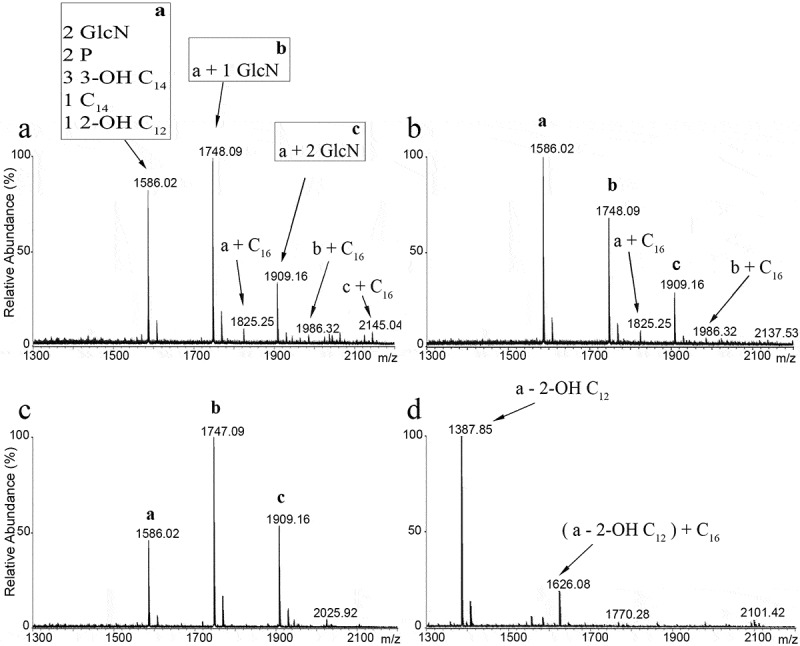
LC-MS analysis of *B. bronchiseptica* lipid A. Comparison of lipid A species from *B. bronchiseptica* strains BB-P19 (a) and BB-D09-SR (b), and the *pagP* (c) and *lpxL1* (d) mutant derivatives, respectively. Major peaks at *m/z* 1586, 1748 and 1909 were interpreted as the characteristic penta-acylated bis-phosphorylated species, and the corresponding species with one and two glucosamine substituents, respectively. Predicted divergence for the other peaks is indicated

As strain-dependent variation in lipid A structure may occur, we determined the structure of lipid A in the two *B. bronchiseptica* isolates that are routinely used in our laboratories. One of these strains was isolated from swine (strain BB-P19) and the other from dog (strain BB-D09-SR). Bacteria were grown under conditions in which they are expected to express the virulent Bvg^+^ phase. The lipid A from these strains was isolated and analyzed by LC-MS. The spectra were similar for both isolates showing three major peaks at *m/z* 1586, 1748 and 1909 (Figure 2a,b). These peaks were attributed to the penta-acylated bis-phosphorylated species in which the lipid A backbone was substituted with three 3‐OH C_14_, one C_14_, and one 2‐OH C_12_ fatty acids (*m/z* 1586), with additional glucosamine substitutions on either one or both phosphate groups (*m/z* 1748 and 1909, respectively). Three additional minor peaks corresponding to the palmitoylated (C_16_) form of the aforementioned species were also present at *m/z* 1825, 1986 and 2145. These hexa-acylated forms represent a conjoined relative abundance of around 10% relative to the penta-acylated species. These results are consistent with the structure shown in Figure 1 and demonstrate that PagP-mediated acylation of lipid A is a minor event in our strains under the applied growth conditions, whilst substitution of the phosphate groups with glucosamine is rather abundant. The LpxO-mediated β-hydroxylation of the secondary acyl chain at the 2 position appears to be essentially complete.

### *Inactivation of* lpxL1 *and* pagP *and effects on lipid A structure*

With the aim of reducing the number of secondary acyl chains in the lipid A structure of *B. bronchiseptica*, we inactivated the *lpxL1* and *pagP* genes (corresponding with locus tags BB0194 and BB4181, respectively, in reference strain RB50). Repeated attempts to similarly inactivate the *lpxL2* gene (locus tag BB0193) failed (data not shown), indicating that this gene is essential in *B. bronchiseptica*, which is consistent with previous observations in *B. pertussis* [12,30]. As expected, LC-MS analysis of lipid A from the *pagP* mutant strain presented the three major peaks also observed in the wild type (*m/z* 1586, 1748 and 1909) but the absence of the corresponding three palmitoylated species (*m/z* 1825, 1986 and 2145) (Figure 2c). Inactivation of *lpxL1* produced substantial differences in the spectrum compared to the wild-type strain (Figure 2d). The predominant peak at *m/z* 1388 corresponds to the molecular ion at *m/z* 1586 of wild-type lipid A with the expected loss of the 2-OH C_12_ chain. However, the expected additional peaks for the corresponding glycosylated forms are missing, suggesting that the LgmB-mediated addition of glucosamines is dependent on the presence of the secondary 2-OH C_12_ residue at the 2 position of the lipid A structure. The additional peak at *m/z* 1626 in the spectrum from the *lpxL1* mutant (Figure 2d) corresponds to the palmitoylated equivalent of the *m/z* 1388 ion. The relative abundance of the palmitoylated form, i.e. ~20%, was higher than in the wild type. Thus, *lpxL1* inactivation results in the loss of the secondary 2-OH C_12_ chain at the 2 position, but this modification also elicits loss of the glucosamine decorations as well as over-palmitoylation.

**Figure 3.**
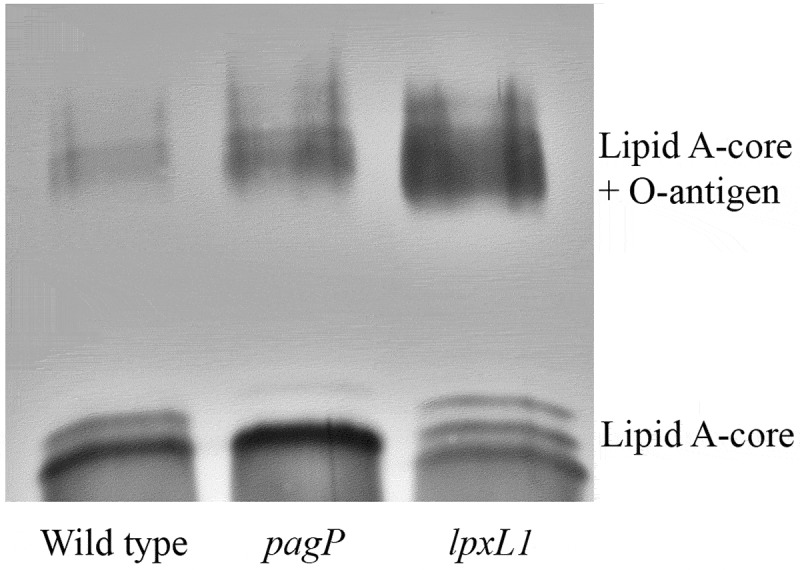
Analysis of LPS modifications by SDS-PAGE. Purified LPS of *B. bronchiseptica* strain BB-D09-SR and of the *pagP* and *lpxL1* mutants was analyzed by SDS-PAGE and visualized by silver staining. The identity of the various bands detected at the position of lipid A plus core sugars is discussed in the text. O-antigen-containing LPS appears as diffuse smear with lower electrophoretic mobility

Purified LPS of the wild type and the mutants was also analyzed on SDS-PAGE gels, showing differences in banding patterns (Figure 3). At the position of LPS molecules consisting of lipid A and core sugars, the wild-type strain showed two bands presumably corresponding to the penta- (lower and more intense band) and hexa-acylated (upper and less intense band) forms and, accordingly, the upper band was completely absent in the LPS of the *pagP* mutant (Figure 3). LPS from the *lpxL1* mutant featured three bands, a lower band, probably corresponding to the LPS with the tetra-acylated lipid A that has lost the 2‐OH C_12_ fatty acid, a band that could correspond with the former but including palmitoylation, and a third band that migrated more slowly on gel than the bands observed in the wild type. As we could not correlate the latter band with any of the peaks observed in the MS analysis, it probably represents LPS molecules with a substitution in the core moiety, perhaps the pentasaccharide linker that connects the O-antigen to the core [31]. In all samples, we also observed complete LPS molecules substituted with O-antigen, but considerably increased amounts of O-antigen-bearing LPS appear to be present in the *lpxL1* mutant (Figure 3).

### TLR4 activation by purified LPS and whole cells

**Figure 4.**
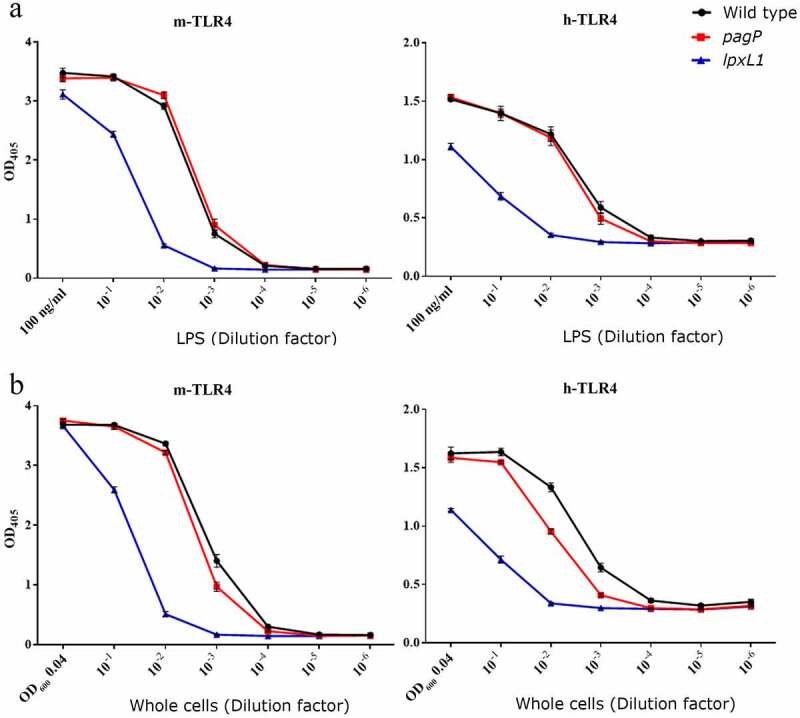
TLR4 activation by purified LPS and whole-cell preparations of strain BB-D09-SR and its *pagP* and *lpxL1* mutant derivatives. HEK-Blue cells expressing either m-TLR4 (left panels) or h-TLR4 (right panels) were incubated for 17 h with 10-fold serial dilutions of (a) purified LPS or (b) heat-inactivated whole cells. The concentrations of the undiluted samples are indicated in ng/ml and OD_600_ units in panels A and B, respectively. Graphs show mean ± SEM of SEAP activity measured at OD_405_ from the supernatants of three independent experiments performed in duplicate. In the stimulation assays with LPS, statistical comparison showed significant differences relative to the wild type only for the *lpxL1* mutant (*P* < 0.0001 for both m- and h-TLR4). When stimulated with whole cells, significant differences were found for the *lpxL1* mutant (*P* < 0.0001 for both m- and h-TLR4) but also for the *pagP* mutant (*P* < 0.05 for m-TLR4 and *P* < 0.0001 for h-TLR4)

To determine whether the modifications in the lipid A structure resulting from the inactivation of *pagP* and *lpxL1* can reduce the endotoxic activity of the LPS of *B. bronchiseptica*, TLR4 activation was tested in HEK-Blue reporter cells expressing either human or murine TLR4 (h- and m-TLR4, respectively) (Figure 4a). Both h- and m-TLR4 were activated to similar levels by LPS from the *pagP* mutant and from the wild-type strain. In contrast, purified LPS of *lpxL1* mutant strain showed significantly lower activity, with 10- to 100-fold higher concentrations being required to reach a similar signal as with the wild-type LPS (Figure 4a).

Our eventual goal is to produce novel whole-cell vaccines with reduced endotoxicity, but reduced endotoxicity of purified LPS preparations is not necessarily reflected in that of whole-cell preparations [15]. Therefore, we also tested the TLR4-stimulating activity of heat-killed whole-cell suspensions with the HEK-Blue cells. As observed with purified LPS, whole cells of the *lpxL1* mutant had a clearly reduced ability to activate m- and h-TLR4 (Figure 4b). Thus, consistent with the results obtained with purified LPS, whole-cell preparations of the *lpxL1* mutant showed reduced TLR4-stimulatory activity. Interestingly, also whole-cell preparations of the Δ*pagP* strain showed a slight, but statistically significant decrease in TLR4 activation compared to wild-type cells (Figure 4b). When we analyzed the LPS content of these cell preparations by SDS-PAGE, we noticed the presence of only a single band representing the lipid A substituted with core sugars in the wild-type cells, and this band migrated more slowly in the gel than the corresponding band in the *pagP* mutant (Figure S1). This result can be explained by assuming that PagP is activated during the heat treatment that is used to kill the cells, resulting in fully palmitoylated LPS. Thus, also the presence of a secondary C_16_ chain bound to the primary 3OH-C_14_ at the 3ʹ position can contribute to the TLR4-stimulating activity of *B. bronchiseptica* LPS.

### *Inactivation of* lpxL1 *reduces the immune response in porcine macrophages*

**Figure 5.**
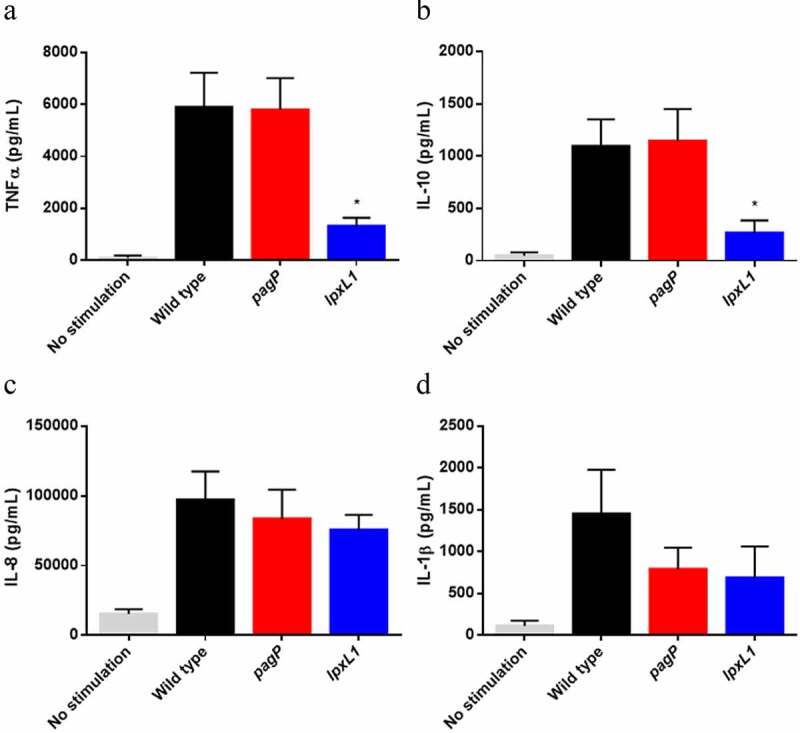
Cytokine secretion upon stimulation of PBMMs with LPS from the *B. bronchiseptica* strain BB-D09-SR and its *pagP*- and *lpxL1*-mutant derivatives. Secreted levels of TNF-α, IL-10, IL-8 and IL-1β by PBMMs of separate porcine individuals (n = 5) were measured after 24 h incubation with 10 ng/ml of purified LPS. Values shown are means and standard deviations from three independent experiments. Statistically significant differences compared to the wild type are indicated with asterisks (*, *P* < 0.05)

As swine livestock is one of the targets for a vaccine against *B. bronchiseptica* and the innate immune system of pigs might respond differently from that of humans and mice, we also investigated the immune response of PBMMs stimulated with LPS. To this end, PBMMs were incubated with purified LPS, and the production of several cytokines (TNFα, IL-10, IL-8, IL-1β and IL-6) was measured. In all cases, the IL-6 concentration was under the detection limit of the technique (data not shown). Stimulation of the PBMMs with LPS from the wild type or the *pagP* mutant resulted in similar production levels of the other cytokines quantified (Figure 5). Although the concentration of IL-1β produced appeared lower upon incubation with LPS from Δ*pagP* strain (Figure 5d), this difference was not statistically significant (*P* = 0.2188). In contrast, PBMMs incubated with LPS from the *lpxL1* mutant produced significantly lower levels of TNFα and IL-10 (Figure 5a,b). Also the IL-1β concentration was lower (Figure 5d), yet not statistically significant (*P* = 0.0663). Production of IL-8 did not differ from the levels induced by wild-type LPS (Figure 5c). This data indicates that the reduced TLR4 activation observed by inactivation of *lpxL1* is consistent in all models tested here.

### Outer membrane permeability of the mutants

**Figure 6.**
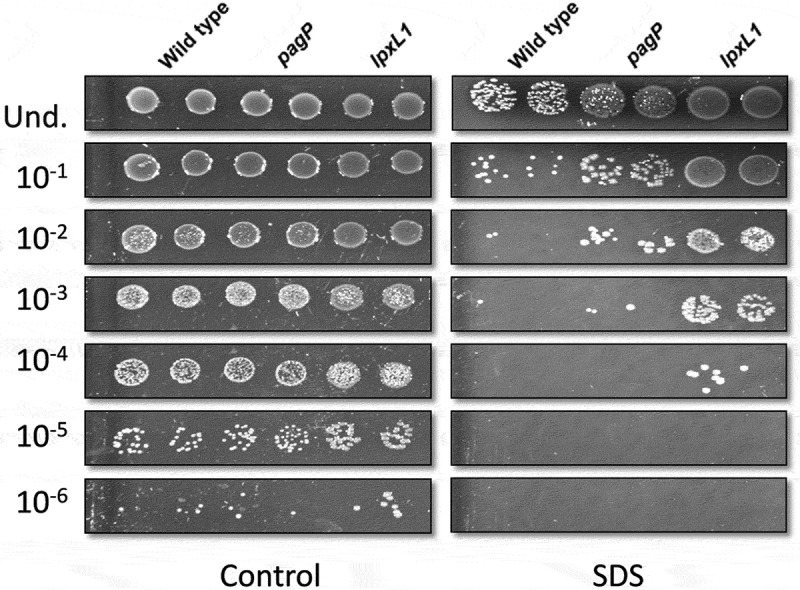
Bacterial sensitivity to SDS. Cultures of strain BB-D09-SR and its *pagP*- and *lpxL1*-mutant derivatives were incubated in duplicate with 1% SDS. After 2 h incubation, drops of 10-fold serial dilutions from these cultures were plated on BG-blood agar and incubated for 48 h. The control at the left shows the results for bacteria not exposed to SDS. A representative result of three independent experiments is shown

The outer membrane of Gram-negative bacteria protects these bacteria by forming a barrier for noxious compounds in the environment, including, for example, alkyl sulfates, which are anionic surfactants [32]. Modifications in the lipid A structure may affect this barrier function. To test whether the *pagP* and *lpxL1* mutations affect membrane permeability, cultures were treated with the anionic surfactant SDS. Survival of the wild-type strain was drastically reduced upon incubation with SDS compared to the untreated control (Figure 6). Interestingly, the *pagP* and *lpxL1* mutants were more resistant to SDS as they showed 10-fold and 1000-fold increased survival, respectively, compared to the wild type (Figure 6). Thus, the mutations fortify the outer membrane of *B. bronchiseptica* by providing an improved barrier to SDS.

Additionally, we tested if the susceptibility of the mutants to different antibiotics and cationic antimicrobial peptides (CAMPs) was affected (Table 1). The susceptibility of the Δ*pagP* strain was similar to that of its parental strain for all antimicrobials tested, except perhaps for polymyxin B that could be slightly decreased. On the other hand, the *lpxL1* mutant was less susceptible to the amphipathic antibiotic ciprofloxacin and it was resistant to the hydrophobic antibiotic rifampicin. In contrast, its susceptibility to polymyxin B and colistin, which are positively charged peptides that bind to the outer membrane of Gram-negative bacteria through electrostatic interactions, was increased. Its resistance to antibiotics that usually are ineffective against Gram-negative bacteria due to their large molecular size, i.e. the glycopeptide vancomycin and the lipopeptide daptomycin, remained unchanged.

**Table 1.** Antimicrobial susceptibility of strain BB-D09-SR and its mutant derivatives^a.^

	Wild type	*pagP*	*lpxL1*
Polymyxin B	0.125	0.125–0.19	0.064–0.125
Colistin	0.125	0.125	0.047–0.094
Ciprofloxacin	0.19	0.19	0.38
Rifampicin	3–4	3–4	>32
Vancomycin	>256	>256	>256
Daptomycin	>256	>256	>256

^a^MICs reported are in μg/ml. Results were obtained in at least three independent experiments. In cases of discrepancy, data was inserted as range.

### *Inactivation of* lpxL1 *reduces cell-surface hydrophobicity and affects biofilm formation*

**Figure 7.**
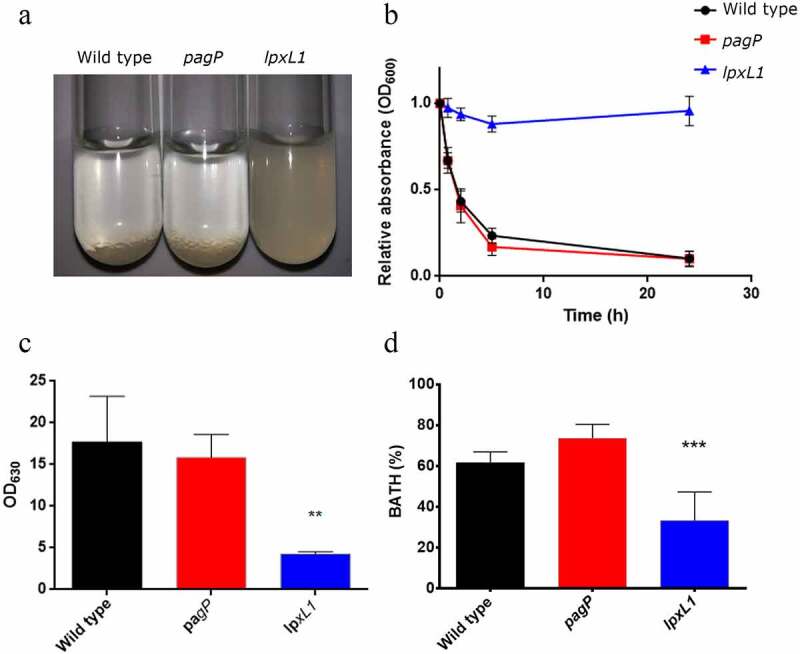
Inactivation of *lpxL1* reduces settling, biofilm formation and surface hydrophobicity. (a) Macroscopic view of bacterial settling. Cultures of strain BB-D09-SR and its mutant derivatives were grown in SS medium supplemented with casamino acids and adjusted to an OD_600_ of 1. A photograph was taken after 5 h incubation under static conditions. (b) Absorbance was measured from the same cultures as shown in panel A. Samples were taken at 0 and 45 min, and at 2, 5 and 24 h. Graph shows mean ± SEM of absorbance relative to the t = 0 sample calculated from four independent experiments. Comparison with the parental strain showed statistically significant differences only for *lpxL1* mutant (*P* < 0.0001). (c) Biofilm formation under static conditions. After 24 h of incubation in SS medium supplemented with casamino acids, biofilms were stained with crystal violet and quantified by measuring the OD_630_. Data represent means and standard deviations from three experiments performed in triplicate. Statistically significant difference compared to the wild type is indicated with asterisks (**, *P* < 0.01). (d) Surface hydrophobicity assessment using BATH method. Bacterial suspensions standardized at OD_600_ of 1 in PBS were mixed with hexadecane. Percentage hydrophobicity was calculated from OD measurements of samples from the water phase. The means and standard deviations from three experiments performed in duplicate are shown. Statistically significant difference compared to the wild type is indicated with asterisks (***, *P* < 0.001)

During our experiments, we noticed that the wild-type *B. bronchiseptica* cells formed aggregates that rapidly settled when the cultures where left standing under static conditions (Figure 7a). This effect was observed during growth in SS medium and, even more prominently, when SS was supplemented with casamino acids, but much less so in Verwey medium. Remarkably, this settling was not observed for the *lpxL1* mutant (Figure 7a). Monitoring of the OD_600_ of the top fractions of the bacterial suspensions confirmed these observations. Already after 45 min, a notable drop in the OD_600_ of the wild-type and the *pagP*-mutant cell suspensions was detected, while that of the *lpxL1* mutant remained unaffected even after 24 h (Figure 7b).

Bacterial auto-aggregation is often related to biofilm formation [33]. Indeed, a significant reduction in biofilm formation was observed for the *lpxL1* mutant strain (Figure 7c). Measurement of the OD_600_ of the supernatants obtained from the biofilms showed significantly higher values for the *lpxL1* mutant (data not shown), discarding the possibility that the reduced biofilm formation of this strain is due to lower growth.

Cell-surface hydrophobicity is one of the physicochemical properties that can determine the initial adhesion and aggregation of bacteria [34]. We hypothesized that the increased levels of hydrophilic O-antigen observed in the LPS of the *lpxL1* mutant (Figure 3) could result in reduced cell-surface hydrophobicity and thereby affect auto-aggregation and biofilm formation. Using the BATH method, we observed that the cell-surface hydrophobicity of the *lpxL1* mutant was indeed significantly reduced relative to that of the wild type, whilst that of the *pagP* mutant was unaffected (Figure 7d).

### Influence of LpxL1 and PagP activity on the infection of macrophages

**Figure 8.**
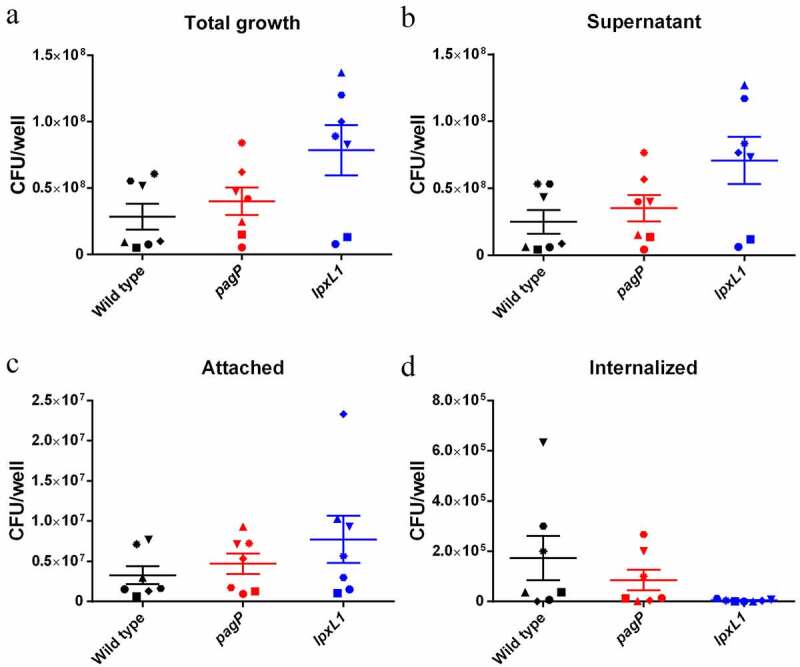
Bacterial survival with pro-inflammatory M1 macrophages. Suspensions of strain BB-D09-SR and its mutant derivatives were incubated with M1 macrophages at an MOI of 1 for 4 h, and CFU in different fractions were quantified. (a) Total bacterial growth (i.e. both inside and outside of the macrophages) expressed as CFU. (b) CFU in the supernatant. (c) CFU attached at the macrophage surface. (d) CFU inside macrophages. Symbols with different shapes correspond to PBMMs of separate porcine individuals (n = 7). No statistical significance was found

*Bordetella* spp. have been described to survive inside professional phagocytes [35,36]. We tested whether the *lpxL1* and *pagP* mutations affect the survival of the bacteria when they are incubated with porcine macrophages. We used both M1 macrophages, which are pro-inflammatory and responsible for inflammatory signaling, and M2 macrophages, which are anti-inflammatory and participate in the resolution of the inflammatory process. The *lpxL1* mutant showed a slight increase in growth when incubated with M1 macrophages, while the *pagP* mutant seemed unaffected relative to the wild type (Figure 8a). When the strains were incubated in RPMI medium without macrophages, no growth advantage of the *lpxL1* mutant was detected (data not shown). The increased amount of *lpxL1* bacteria was reflected in the supernatant (Figure 8b) and the adhesion to the macrophage surface (Figure 8c), but in contrast to the wild type, the *lpxL1* mutant was barely detected inside the macrophages (Figure 8d). Nevertheless, none of these differences were statistically significant. Similar tests were performed with M2 macrophages (Supplementary Figure S2), where the reduced infectivity of the *lpxL1* mutant was not observed (Figure S2D).

### *Presence of vaccine antigens in the outer membrane of the* lpxL1*mutant*

**Figure 9.**
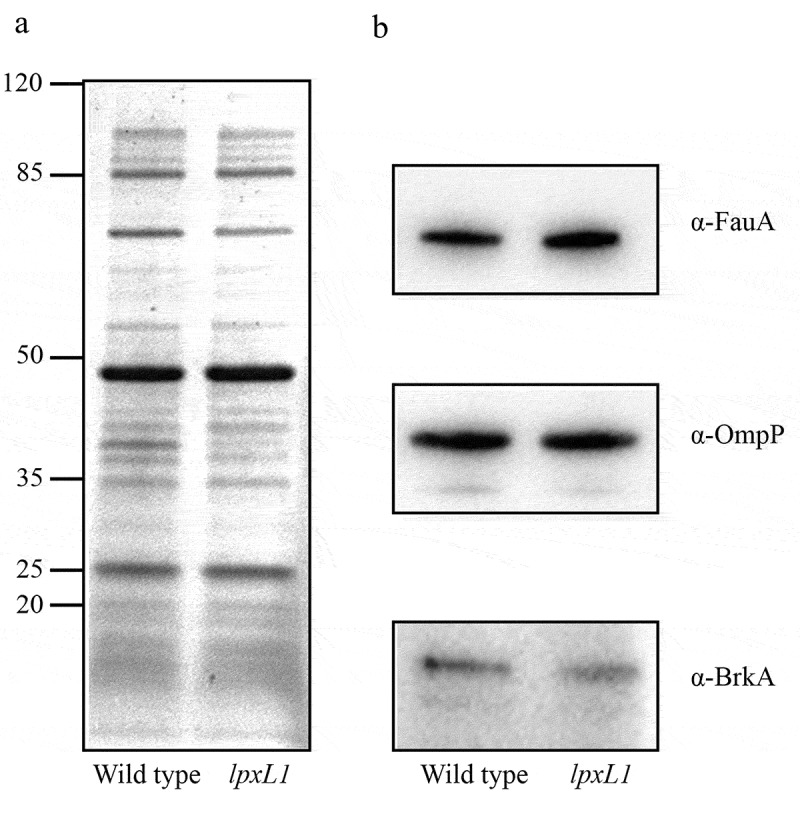
Comparison of protein content of outer-membrane preparations from *B. bronchiseptica* strain BB-D09-SR and its *lpxL1* mutant derivative. (a) SDS-PAGE analysis of isolated outer membranes of the wild-type strain and its *lpxL1* mutant derivative. Molecular weight markers are shown at the left. (b) Western blot analysis of relevant outer membrane antigens. Membranes were incubated with antibodies directed against the siderophore receptor FauA, major porin OmpP, and autotransporter BrkA

As the LPS modifications in the *lpxL1* mutant could potentially affect the localization of relevant protective antigens at the cell surface, we compared the protein profiles of outer membranes isolated from the parental strain and from its *lpxL1* mutant derivative. With the exception of an unidentified protein of ~40 kDa, which was present in diminished amounts in the *lpxL1* mutant, the protein profiles were almost identical (Figure 9a). In addition, the presence of several proteins that are considered as potential vaccine antigens, i.e. the iron-regulated siderophore receptor FauA [37,38] the major porin OmpP [39], and the *Bordetella* resistance-to-killing autotransporter BrkA [40], was investigated by Western blotting. All these proteins were equally well detectable in the outer-membrane preparations of both strains (Figure 9b).

## Discussion

Modifications in the structure of the lipid A are associated with changes in the endotoxicity of the LPS, which can be used to develop less reactogenic whole-cell vaccines. In *B. bronchiseptica*, PagP and LpxL1 function as acylases attaching secondary acyl chains to primary fatty acids in the lipid A. For PagP, this function has previously been demonstrated in *B. bronchiseptica* [13], but the function of LpxL1 has only been validated in *B. pertussis*. In *B. pertussis, lpxL1* is normally not expressed under laboratory conditions, yet its overproduction resulted in secondary acylation [12]. The consequences of *pagP* and *lpxL1* expression on the TLR4-stimulating activity of LPS of *B. bronchiseptica* were still unknown. Our mass spectrometry analysis confirmed the non-stoichiometric PagP-mediated palmitoylation in only ~10% of the molecules in our wild-type strains, the complete absence of this palmitoylation in a *pagP* mutant, and the loss of secondary 2-OH C_12_ in the 2 position in an *lpxL1* mutant. In the latter mutant, palmitoylated lipid A increased up to ~20% and, interestingly, we also observed that the abundant glucosamine substitution at the phosphate groups detected in our wild-type strains was fully abolished in the *lpxL1* mutant. This suggests that the absence of the secondary 2-OH C_12_ makes this structure a poor substrate for the introduction of glucosamines by the LgmB glycosyltransferase. A similar defect was observed in *E. coli* and *Salmonella* Typhimurium, where inactivation of *lpxM*, which mediates insertion of a secondary myristate group at the 3ʹ position, resulted in the loss of L-Ara4N addition to the phosphate groups of their lipid A [41]. Moreover, glucosamine substitutions are rarely found in *B. pertussis* despite the presence of an intact *lgmB* locus [16], which might be due to the lack of LpxL1 activity. Additionally, inactivation of *lpxL1* resulted in an increased O-antigen substitution in the LPS (Figure 3).

Reduction of the number of acyl chains in lipid A is usually related to lower activation of TLR4 and, therefore, lower endotoxicity [42]. We showed here that inactivation of *pagP* did hardly or not affect TLR4 activation or any other phenotype of the cells tested. Since palmitoylation of LPS by PagP was quite low in our wild-type strains (Figures 2 **and** 3), it could be that the difference between wild type and mutant is too small to allow for a substantial effect on TLR4 activation. However, analysis of the LPS of the heat-killed cells that were used in the TLR4 activation assays by SDS-PAGE indicated that the LPS in the wild-type samples is fully palmitoylated (Figure S1), suggesting that PagP is activated during the heat treatment. This hypothesis is in line with our previous studies in which we used the same heat treatment to stimulate the release of outer-membrane vesicles (OMVs) [29]. Lipidomic analysis showed a large increase in lysophospholipid content in heat-released OMVs compared to spontaneously released OMVs [43]. As this increase in lysophospholipid content was reduced when OMVs were isolated from a *pldA* mutant, it can be explained in part, but not completely, by the activation of the outer-membrane phospholipase A, which is encoded by the *pldA* gene, during the heat treatment. It was suggested that the remaining lysophospholipids could possibly be the result of activation of PagP [43]. The high level of palmitoylation of LPS in the heat-killed cells, suggested by the results in Figure S1, is consistent with this hypothesis. In addition, this explains the slightly, but significantly reduced TLR4 activation of the *pagP* mutant when heat-killed whole cells were used as TLR4 agonist (Figure 4b), but not when purified LPS was used (Figure 4a).

We observed a strong reduction of TLR4 signaling upon inactivation of *lpxL1*. In porcine macrophages, we observed a significant decrease in the production of the pro-inflammatory cytokine TNFα, but also of the anti-inflammatory cytokine IL-10. Induction of the latter occurs concomitantly with the pro-inflammatory cytokines upon LPS-induced TLR4 activation, and its role is to provide immediate feedback to limit the immune response to pathogens [44]. In our case, the reduced stimulation of TLR4 appears to influence both pro- and anti-inflammatory pathways. The main lipid A species in this *lpxL1* mutant is tetra-acylated but also non-glycosylated. Whether the reduced endotoxicity is a consequence of the loss of the acyl chain, the glucosamines, or both is unknown. In a previous study, *lgmB* inactivation in *B. bronchiseptica* showed no effect on TNFα production in murine macrophages [17]. However, the level of glucosamine substitutions in the wild-type strain used in that study appears to be rather low compared to that in the strains used in our study, which could be a reason for the absence of an effect of the *lgmB* mutation on TLR4 activation. Other studies performed in *B. pertussis* and *Bordetella parapertussis* have demonstrated that the substitution of the phosphate groups with glucosamine increases the TLR4-activating capacity. [45],showed that inactivation of *lgmB* in a *B. pertussis* strain with a low level of glucosamine substitution resulted in a small but statistically significant reduction in IL-6 production by human monocytes, whilst inactivation of *lgmB* in a *B. parapertussis* strain with a much higher level of lipid A glycosylation affected IL-6 production more drastically. Consistently, [19],reported that inactivation of *lgmB* in a *B. pertussis* strain with a high abundance of glucosamines in the lipid A drastically reduced h-TLR4 activation. Interestingly, the *lgmB* mutation in that study did not affect m-TLR4 activation, suggesting species specificity in the role of the glucosamines. In our study, the reduced TLR4 activation by the *lpxL1* mutant was detected with both h- and m-TLR4 reporter cells, as well as with porcine macrophages. This difference indicates that the loss of the 2-OH C_12_ chain in the *lpxL1* mutant contributes to the reduced activity observed at least for the m-TLR4. Alternatively, the glucosamines could have a different impact on the LPS activity in *B. bronchiseptica* compared to *B. pertussis*.

It is important to note that the inactivation of *lpxL1* in *B. bronchiseptica* led to reduced TLR4 activation by both purified LPS and whole-cell preparations. An identical tetra-acylated lipid A structure was obtained by the heterologous expression of the de-acylase PagL in *B. pertussis* [15]. The resulting LPS showed reduced TLR4 activation in vitro, but whole cells of the recombinant strain showed even higher activity than wild-type cells [15]. This was reported to be due to the increased release of the tetra-acylated LPS from the cell surface in *B. pertussis* and the higher potency of released LPS relative to membrane-bound LPS in activating TLR4. Apparently, the LPS in the *B. bronchiseptica lpxL1* mutant is still stably anchored in the outer membrane.

The *lpxL1* mutant generated showed many pleiotropic phenotypes, including a less hydrophobic cell surface probably due to a raised portion of LPS molecules bearing O-antigen. Possibly, the under-acylated and non-glycosylated structure is less efficiently recognized by the Lpt machinery, which mediates the transport of the LPS molecules to the outer membrane [46]. The prolonged residence time at the periplasmic leaflet of the inner membrane might allow the WaaL ligase to substitute the lipid A-core subunits more efficiently with O-antigen. This change is possibly responsible for the reduced bacterial auto-aggregation and biofilm formation observed. We noticed an increased susceptibility of the *lpxL1* mutant to CAMPs. This phenotype is presumably associated with the rise of negative charges in the lipid A as a result of the loss of glucosamine decorations and is in agreement with previous studies which showed that inactivation of *lgmB* increases susceptibility to CAMPs in *B. bronchiseptica* [17] and *B. pertussis* [47]. Additionally, the reduced number of acyl chains might be contributing to the increased susceptibility to CAMPs, as increased acylation has previously been reported to represent a defense mechanism against CAMPs in other species [48]. The more negatively charged LPS binds more divalent cations, which results in an increased integrity of the outer membrane and, therefore, a reduction of permeability to amphipathic and hydrophobic antimicrobials [49], as evinced here by the reduced susceptibility to SDS and ciprofloxacin, and rifampicin, respectively. In addition, the increased amount of O-antigen could be boosting the protection against these antimicrobials.

LPS can facilitate adhesion, but it can also act as physical impediment for other adhesins [50,51]. Inactivation of *lpxL1* did not affect adhesion of the bacteria to pro-inflammatory porcine macrophages, but the infection of these cells appeared to be reduced although the difference was not significantly different (Figure 8). This observation is consistent with a previous report which showed that inactivation of *lpxL1* in *B. pertussis* impaired infection of human macrophages although adhesion was even enhanced [12]. We found a substantial reduction of TLR4 activation by the *lpxL1* mutation, which could lead to downsized phagocytosis [52,53] and may explain the reduced numbers of bacteria detected inside macrophages. However, this explanation cannot hold for *B. pertussis*, where LpxL1 does not acylate lipid A during in vitro growth and, hence, its inactivation cannot affect TLR4 activation. Therefore, the reduced infection of human macrophages by the *B. pertussis lpxL1* mutant suggests *lpxL1* expression is induced inside the macrophages and is important for intracellular survival.

Overall, our results point at the *B. bronchiseptica lpxL1* mutant as a potential candidate for the development of novel whole-cell vaccines with reduced reactogenicity. In addition, this strain presents increased amounts of O-antigen, which can be beneficial for an enhanced immune protection [54]. Furthermore, we demonstrated that the outer-membrane protein profile is barely affected by the mutation, and we confirmed the presence of several potential vaccine antigens at similar levels in the outer membrane of the mutant as in the wild type, which suggests its capability of inducing an appropriate immune response. In future studies, we will investigate the reactogenicity of the modified bacteria in vivo and study the protective immunity induced in target animals.

## Supplementary Material

Supplemental Material

## Data Availability

The data used and/or analyzed during the current study are available from the corresponding author on reasonable request.
