# Supplementary material for: Reduction of endotoxicity in Bordetella bronchiseptica by lipid A engineering: Characterization of lpxL1 and pagP mutants
Source: Virulence. 2021 May 31;12(1):1452–68. doi: 10.1080/21505594.2021.1929037 (PMC8168481; doi:10.1080/21505594.2021.1929037)
Supplement: Supplemental Material [file KVIR_A_1929037_SM1149.zip › Supplemental material Revised.docx]

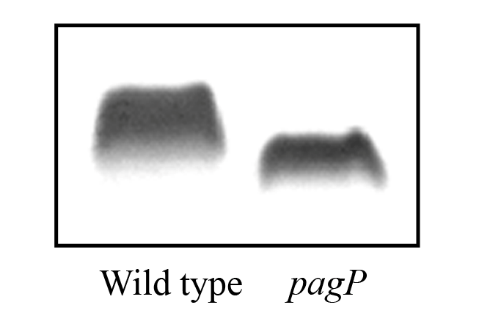
**Figure S1 |** Analysis of LPS in heat-killed whole-cell preparations. Whole-cell lysates of heat-killed *B. bronchiseptica* strain BB-D09-SR and its *pagP* mutant derivative were analyzed by SDS-PAGE, and LPS was stained with silver. Only the part of the gel showing the position of lipid A plus core sugars is shown.


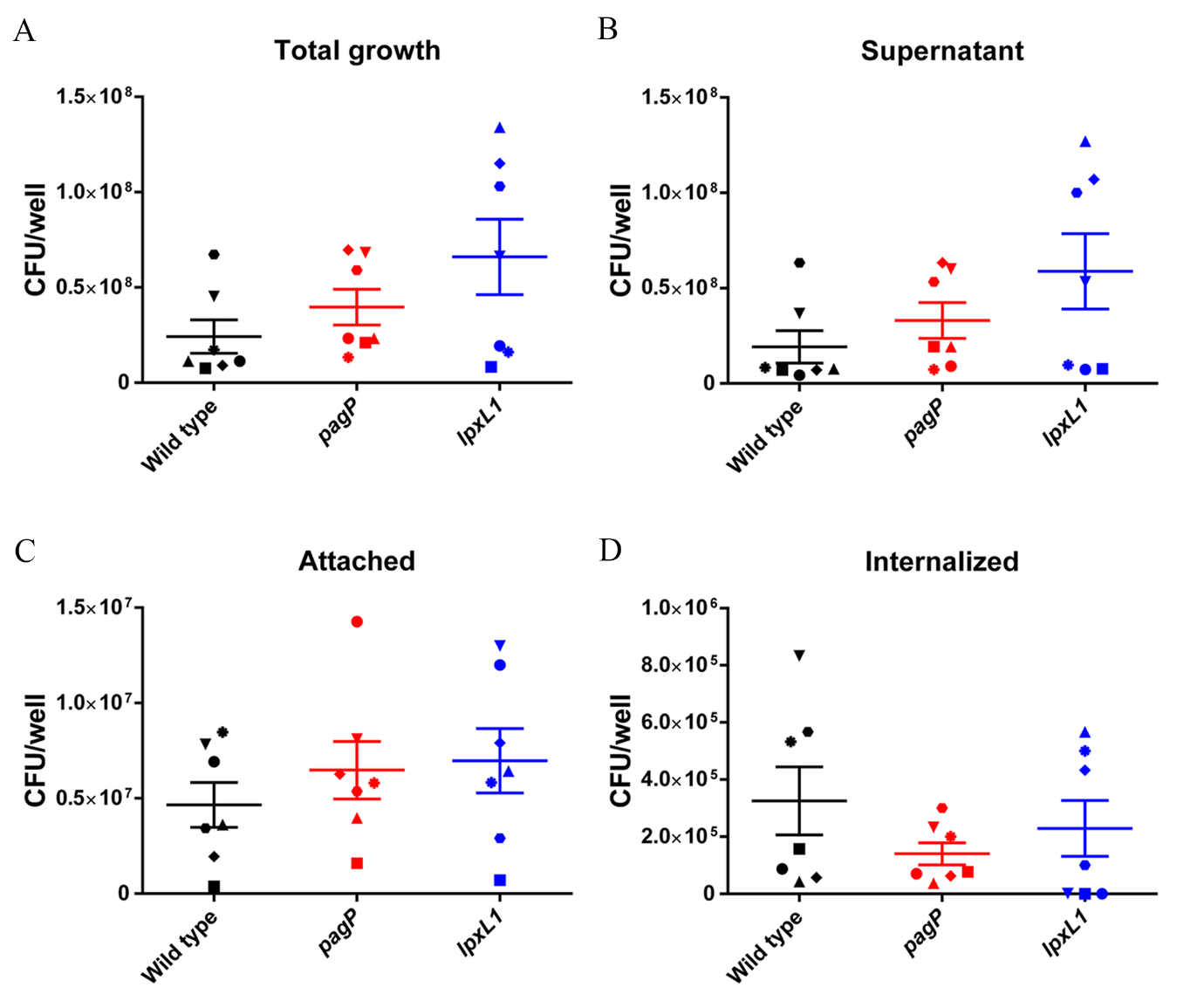


**Figure S2** **|** Bacterial survival with anti-inflammatory M2 macrophages. Suspensions of strain BB-D09-SR and its derivatives were incubated with M2 macrophages at an MOI of 1 for 4 h, and CFU in different fractions were quantified. **(A)** Total bacterial growth (i.e. both inside and outside the macrophages) expressed as CFU. **(B)** CFU in the supernatant. **(C)** CFU attached at the macrophage surface. **(D)** CFU inside macrophages. Symbols with different shapes correspond to PBMMs of separate porcine individuals (n=7). Data was analyzed for statistical significance using one-way ANOVA with repeated measures (Dunnett’s correction for multiple comparison). No significant differences were found.

**Table S1 |** Bacterial strains and plasmids

| Strain / plasmid | Description ^a^ | Reference ^b^ |
| --- | --- | --- |
| *B. bronchiseptica* |  |  |
| BB-P19 | Clinical isolate from pig  (i. n. 2130108058), Cef^R^, Str^R^ | UU |
| BB-D09 | Clinical isolate from dog  (i. n. 2170524052), Cef^R^ | UU |
| BB-D09-SR | Spontaneous Str^R^ derivative of BB-D09 | This study |
| BB-P19 Δ*pagP* | *pagP* mutant of BB-P19, Cef^R^, Str^R^, Gem^R^ | This study |
| BB-D09-SR Δ*pagP* | *pagP* mutant of BB-D09-SR, Cef^R^, Str^R^, Gem^R^ | This study |
| BB-D09-SR *lpxL1* | *lpxL1* mutant of BB-D09-SR, Cef^R^, Str^R^, Gem^R^ | This study |
| *E. coli* |  |  |
| DH5α | F^−^, Δ(*lacZYA-argF)U169 thi-1 hsdR17 gyrA96 recA1 endA1 supE44 relA1 phoA Φ80 dlacZΔM15* | (Grant et al., 1990) |
| SM10(λpir) | *thi thr leu fhuA lacY supE recA*::RP4-2-Tc::Mu *λpir* R6K. Kan^R^ | (Simon et al., 1983) |
| *Plasmids* |  |  |
| pCRII | *E. coli* cloning vector, Amp^R^, Kan^R^ | Invitrogen |
| pYRC | pBBR1MCS-5 *lacI* cloning vector, Gem^R^ | (Arts et al., 2007) |
| pCRII-PagPup | pCRII derivative harboring *pagP*-upstream sequence, Amp^R^, Kan^R^ | This study |
| pCRII-PagPdw | pCRII derivative harboring *pagP*-downstream sequence, Amp^R^, Kan^R^ | This study |
| pCRII-PagPup-PagPdw | pCRII derivative harboring *pagP*-up/down-stream sequence, Amp^R^, Kan^R^ | This study |
| pCRII-PagPup-GemR-PagPdw | pCRII-PagPup-PagPdw harboring gentamicin-resistance cassette, Amp^R^, Kan^R^, Gem^R^ | This study |
| pCRII-upLpxL1dw | pCRII derivative harboring *lpxL1*-locus and up/down-stream sequence, Amp^R^, Kan^R^ | This study |
| pCRII-upLpxL1dw-GemR | pCRII-upLpxL1dw harboring gentamicin-resistance cassette, Amp^R^, Kan^R^, Gem^R^ | This study |
| pKAS32 | Allelic exchange suicide vector, Amp^R^ | (Skorupski and Taylor, 1996) |
| pKAS-PagPup-GemR-PagPdw | pKAS32 derivative harboring *pagP* knockout construct, Amp^R^, Gem^R^ | This study |
| pKAS-upLpxL1dw-GemR | pKAS32 derivative harboring *lpxL1* knockout construct, Amp^R^, Gem^R^ | This study |

^a^ Cef, cefotaxime; Str, streptomycin; Gem, gentamicin; Amp, ampicillin; Kan, kanamycin; i. n., isolate number.

^b^ UU, Veterinary Microbiological Diagnostic Centre, Division Infectious Diseases & Immunology, Faculty of Veterinary Medicine, Utrecht University

**References**

Arts, J., van Boxtel, R., Filloux, A., Tommassen, J., and Koster, M. (2007). Export of the pseudopilin XcpT of the *Pseudomonas aeruginosa* type II secretion system via the signal recognition particle-Sec pathway. *J. Bacteriol.* 189, 2069–2076. doi:10.1128/JB.01236-06.

Grant, S. G. N., Jessee, J., Bloom, F. R., and Hanahan, D. (1990). Differential plasmid rescue from transgenic mouse DNAs into *Escherichia coli* methylation-restriction mutants. *Proc. Natl. Acad. Sci. U. S. A.* 87, 4645–4649. doi:10.1073/pnas.87.12.4645.

Simon, R., Priefer, U., and Pühler, A. (1983). A broad host range mobilization system for in vivo genetic engineering: Transposon mutagenesis in gram negative bacteria. *Bio/Technology* 1, 784–791. doi:10.1038/nbt1183-784.

Skorupski, K., and Taylor, R. K. (1996). Positive selection vectors for allelic exchange. *Gene* 169, 47–52. doi:10.1016/0378-1119(95)00793-8.

**Table S2 |** PCR primers used in this study

| Primer | Sequence (5’🡪3’) ^a^ | Description |
| --- | --- | --- |
| Fw-PagPup | ACAAGCTGCAAGGCGTCCTG | Primers for amplification of region upstream *pagP*, introducing an Eco81I restriction site |
| Rv-PagPup-Eco81I | GCGCGCCCTCAGGGTCATATGCTGCGCTAACGG |  |
| Fw-Eco81I-PagPdw | GCGCGCCCTGAGGTTCATGTTTGGCCGCTGGG | Primers for amplification of region downstream *pagP*, introducing an Eco81I restriction site |
| Rv-PagPdw | CATCGAAGCGGTCGACTTGC |  |
| Fw-Eco81I-GemR | GCGCGCCCTGAGGGACGCACACCGTGGAAA | Primers for amplification of *gem^R^*, introducing Eco81I restriction sites at both flanks |
| Rv-GemR-Eco81I | GCGCGCCCTCAGGGCGGCGTTGTGACAATTT |  |
| Fw-upLpxL1 | AATTCGCTCTGGCGCTGCAC | Primers for amplification of *lpxL1* locus with partial upstream and downstream sequence |
| Rv-LpxL1dw | ATCAGGGCATTGATGCGTTC |  |
| Fw-PfoI-GemR | GCGCGCTCCTGGAGACGCACACCGTGGAAA | Primers for amplification of *gem^R^*, introducing PfoI restriction sites at both flanks |
| Rv-GemR-PfoI | GCGCGCTCCAGGAGCGGCGTTGTGACAATTT |  |

^a^ Restriction sites are underlined
